# A Sensor Network Utilizing Consumer Wearables for Telerehabilitation of Post-Acute COVID-19 Patients

**DOI:** 10.1109/JIOT.2022.3188914

**Published:** 2022-07-07

**Authors:** Miroslav Bures, Katerina Neumannova, Pavel Blazek, Matej Klima, Hynek Schvach, Jiri Nema, Michal Kopecky, Jan Dygryn, Vladimir Koblizek

**Affiliations:** Department of Computer ScienceFaculty of Electrical EngineeringCzech Technical University in Prague 121 35 Prague Czechia; Department of PhysiotherapyFaculty of Physical CulturePalacký University Olomouc 771 47 Olomouc Czechia; Military Medical Management DepartmentFaculty of Military Health SciencesUniversity of Defence 48287 500 01 Hradec Kralove Czechia; Faculty of Medicine in Hradec KraloveCharles University 37740 110 00 Prague Czechia; Institute of Active Lifestyle, Faculty of Physical Culture, Palacký University Olomouc 771 47 Olomouc Czechia; Department of PneumologyUniversity Hospital Hradec Kralove 48234 500 05 Hradec Kralove Czechia

**Keywords:** COVID-19, Internet of Things, personal wearables, sensor network, telecoaching, telerehabilitation

## Abstract

A considerable number of patients with COVID-19 suffer from respiratory problems in the post-acute phase of the disease (the second–third month after disease onset). Individual telerehabilitation and telecoaching are viable, effective options for treating these patients. To treat patients individually, medical staff must have detailed knowledge of their physical activity and condition. A sensor network that utilizes medical-grade devices can be created to collect these data, but the price and availability of these devices might limit such a network’s scalability to larger groups of patients. Hence, the use of low-cost commercial fitness wearables is an option worth exploring. This article presents the concept and technical infrastructure of such a telerehabilitation program that started in April 2021 in the Czech Republic. A pilot controlled study with 14 patients with COVID-19 indicated the program’s potential to improve patients’ physical activity, (85.7% of patients in telerehabilitation versus 41.9% educational group) and exercise tolerance (71.4% of patients in telerehabilitation versus 42.8% of the educational group). Regarding the accuracy of collected data, the used commercial wristband was compared with the medical-grade device in a separate test. Evaluating $z$-scores of the intensity of participants’ physical activity in this test, the difference in data is not statistically significant at level $p=0.05$. Hence, the used infrastructure can be considered sufficiently accurate for the telerehabilitation program examined in this study. The technical and medical aspects of the problem are discussed, as well as the technical details of the solution and the lessons learned, regarding using this approach to treat COVID-19 patients in the post-acute phase.

## I. Introduction

The COVID-19 pandemic, which is close to entering its third year, has brought significant challenges to the global healthcare system. Apart from the considerable difficulties in managing patients in the disease’s acute phase [1], numerous COVID-19 patients who have experienced more serious symptoms require an adequate rehabilitation program in the post-acute phase. After having COVID-19, patients often complain of fatigue, breathing difficulties, and muscle weakness [2], [3]. Moreover, they frequently believe they will be unable to return to their pre-COVID-19 physical activity levels. Therefore, it is optimal for patients to participate in targeted and interactive programs focused on improving their physical activity levels. For these types of rehabilitation programs, knowledge regarding patients’ physical activity, quality of sleep, and other vital function data are helpful for working with individual patients. Moreover, the collected information further allows for the optimization of treatment and rehabilitation procedures.

However, some patients have limited access to outpatient pulmonary rehabilitation programs [4]. Therefore, telerehabilitation may be a suitable solution for these patients. Previous studies have confirmed the positive effect of telerehabilitation and telecoaching on reducing symptom severity, increasing exercise capacity and physical activity, and improving health-related quality of life in patients with chronic obstructive pulmonary disease (COPD) and idiopathic pulmonary fibrosis [5]–[7]. Nevertheless, only a few studies have evaluated the effect of telerehabilitation and telecoaching on functional status in patients with post-acute COVID-19. The results of these studies [8]–[11] also demonstrated the positive effect of telerehabilitation in these patients. Therefore, telerehabilitation, including individualized telecoaching, could be an option for treating patients with post-acute COVID-19, in whom symptoms are present beyond three weeks but less than 12 weeks after disease onset [2], [12].

For optimally guided telecoaching, it is important to determine the intensity and level of daily physical activity. Sensor networks can be very helpful for this purpose; however, considering the number of affected patients, the time needed to develop proprietary hardware for specific cases is limiting, and development and production of specialized hardware can involve high costs. Numerous commercial fitness applications are available for utilize wearable consumer electronics, but only a few allow direct connection with medical staff to optimize rehabilitation procedures for individual patients.

The logical option worth exploring is to employ commercial wearables to collect the required patient data. A lower price and shorter hardware acquisition time represent substantial benefits compared to the proprietary hardware development. Furthermore, in situations where the accuracy of the obtained data is acceptable for the specific case, building a sensor network from commercial wearables is a viable option.

We applied this idea in the recent telerehabilitation self-training assistant (TERESA) project, which focuses on the telerehabilitation in patients with post-acute COVID-19 who have experienced serious symptoms. In this project, symptomatic patients continued their pulmonary rehabilitation program online after an initial face-to-face informational lesson with a physiotherapist. The telerehabilitation lessons also included telecoaching support. Telecoaching support involved wearing a fitness tracking device throughout the day and receiving weekly feedback from the physiotherapist. Previous studies [13]–[15] have shown that access to pulmonary rehabilitation treatment is not easy for many patients due to various reasons, including availability; low treatment referral by healthcare professionals; distance, travel, and transport complications; lack of knowledge of treatment benefits; low motivation; and the small number of therapists specializing in this treatment. Therefore, it is important to find ways to promote more and easier access to rehabilitation treatment, including sufficient support for increasing the daily level of physical activity. We hypothesized that our program would facilitate early and easy participation in the rehabilitation process in the patient’s home environment. Therefore, the main aim of our project was to confirm that easier access to rehabilitation services for patients with problems related to post-acute COVID-19 could lead to improvement in their functional status and overall health.

This study aims to present the telerehabilitation concept and report on its feasibility and preliminary results providing encouragement for evolving the concept further and verifying it in a larger pilot study.

Despite its preliminary nature, this study makes the following contributions.

1) It provides a description of the telerehabilitation and telecoaching concepts for post-acute COVID-19 management.
2) It presents a relatively straightforward and rapid-to-deploy technical approach that can be helpful in COVID-19 management situations that are similar to the discussed case.
3) The results of the initial controlled study verify the approach.
4) The discussion and lessons learned present methods for quickly overcoming various technical obstacles and pitfalls that might occur during the implementation of such a system.

The remainder of this article is organized as follows. Section II discusses the related work. Section III introduces the TERESA project and the telerehabilitation process we implemented. Section IV presents the technical details of the technical infrastructure used in this project. Section V presents the design and results of the pilot study with patients with post-acute COVID-19 and discusses the reliability and accuracy of the collected data from a technical perspective. Section VI summarizes the lessons learned and provides recommendations for those interested in creating a similar system. Section VII discusses the results and possible threats to validity. Section VIII concludes this article.

## II. Related Work

Many examples of sensor networks relevant for COVID-19 management can be found, as studies on the possibilities of using sensors in modern wearables for sports rehabilitation [16], disease diagnosis [17], and health monitoring in general [18]–[21] have been increasing in recent years. Regarding health monitoring, which is a focus in our study, security and privacy issues have been examined [22], [23].

Focusing on the specific area of pulmonary telerehabilitation and telecoaching, electronic devices, information, and communication technologies can offer more possibilities for accessing pulmonary rehabilitation programs and for improving behavior strategies that target achieving sufficient levels of physical activity in chronically ill patients [4], [5].

At present, various activity monitors, such as pedometers, fitness bracelets, smartwatches, and smartphones, can provide people with fast, direct feedback on how active they are. However, the apps for these commercial devices are not able to specify physical activity recommendations for chronically ill patients. If therapists have access to data regarding patients’ steps per day and physical activity intensity, they can set individual recommendations for patients and guide them on how to perform and gradually increase their physical activity. This can be done in in-person meetings during outpatient pulmonary rehabilitation or online meetings during telerehabilitation and telecoaching. The advantage of telerehabilitation and telecoaching is the removal of barriers that may complicate access to outpatient pulmonary rehabilitation [4], [5], [13]–[15]. In addition, the more common and available activity monitors are used, the more likely it is that patients can easily participate in such programs. Moreover, a telecoaching program can be supported by semiautomated applications [5], [24], which can send texts with motivational messages and feedback and revise weekly daily activity goals. Semiautomated telecoaching programs are well accepted and feasible not only for therapists but also for patients with COPD [24]; they have been shown to increase the daily level of physical activity and exercise tolerance in these patients [5]. It can be thus assumed that these programs could be helpful for other chronic diseases as well as in symptomatic patients with post-acute COVID-19 [12].

## III. TERESA Project Concept

The TERESA project focuses on assessing the effect of telerehabilitation with telecoaching on the functional status of patients with post-acute COVID-19, in whom symptoms associated with breathing difficulties and fatigue persist during activities of daily living and/or during common physical exercises. In this project, telerehabilitation with telecoaching began after the initial assessment of the functional status of symptomatic patients with post-acute COVID-19. Patients underwent initial education with a pulmonologist and physiotherapist. Moreover, the physiotherapist taught patients individual exercises that were part of an educational brochure that each patient received for home exercise and breathing training. Furthermore, the pulmonologist and the physiotherapist provided specific guidance for exercise training to patients with post-acute COVID-19 during the educational lesson. The detailed recommendations for exercise training were also written in the educational brochure. Patients were instructed to contact the pulmonologist or the physiotherapist if they experienced worsening dyspnoea, chest or muscle pain, cardiac problems, muscle weakness or difficulty speaking during exercise training, as is recommended in Physiotherapy management of COVID-19 in the acute hospital setting and beyond [3]. The physiotherapist then conducted and supervised telerehabilitation lessons through video calls once per week. Each telerehabilitation lesson consisted of education, an exercise program, personalized patient telecoaching, and a question-and-answer session. The daily level of physical activity was continuously monitored using a smart wristband. The physiotherapist obtained collected data on daily physical activity (number of steps per day and intensity of physical activities) in a weekly report, which were used as input for personalized patient telecoaching. Personalized patient telecoaching was focused on evaluating the level of physical activity in the previous week and the patient was discussed how he should be physically active in the following week. The primary outcome was the daily level of physical activity at post-treatment measured by number of steps per day.

## IV. Technical Details

The consumer wearable used in this study was *Xiaomi Mi Smart Band 5*, hereafter referred to as “Wristband.” In the Wristband, data about user activity are acquired via a PPG Heart rate sensor, 3-axis accelerometer, 3-axis gyroscope, and Proximity sensor. Internal GPS is not integrated into the Wristband so that GPS position can be derived from the GPS position of the mobile phone connected to the Wristband. Bluetooth v5 BLE with Dialog DA14580 chip is used for communication with a mobile phone. The Wristband was accompanied by a set of two mobile apps and a back-end server for data collection. This particular wearable device was selected for the pilot run because of its favorable price-to-value ratio.

**Fig. 1.**
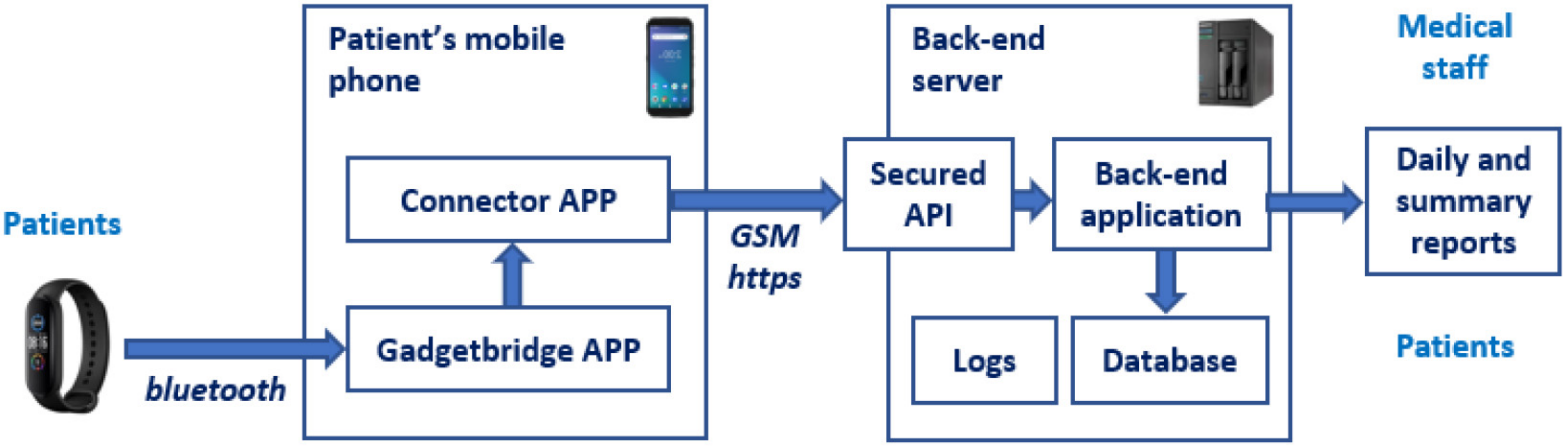
Overall technical schema of the system.

The overall system is shown in Fig. 1. The patient’s Wristband sent the collected data via Bluetooth to the Gadgetbridge^1^ app on the patient’s mobile phone. The data from Gadgetbridge were then passed to the Connector app, our proprietary mobile app handling the communication with the back-end server. This app was also used for interactive communication with patients and to administer questionnaires or collect feedback during the rehabilitation procedure.^1^https://f-droid.org/en/packages/nodomain.freeyourgadget.gadgetbridge/

In the system’s back end, data were received and securely stored. Medical staff could download reports about patients’ daily activity from the secure administrator app.

### A. Collected Patient Data

Collected patient data were derived from the options provided by the Wristband. In this study, we collected: 1) pulse; 2) number of steps per day; and 3) time spent performing physical activity of a particular intensity (see Section IV-B for more details).

The hardware configuration used further allowed patient GPS position to be collected, not taken from the Wristband (which does not have a GPS module) but from the mobile phone used for the data transfer. Moreover, on Wristband’s touch display, the type of physical activity being performed could be specifically selected (e.g., running, cycling, etc.) and then transmitted to the system’s back end for further processing.

The Wristband allowed data to be grouped as 1-min intervals. This sampling rate was maintained throughout the entire data transfer pipeline, and data were saved in the server’s back-end database using 1 min as the smallest measured time period.

Considering the required confidentiality of patient data used for medical purposes, we did not use the Wristband provider’s cloud service to store, aggregate, or download data. Instead, we used a proprietary solution to have more control over the data collection process, as presented in Fig. 1.

### B. Patient Activity Reports

The medical staff evaluated patient activity during the rehabilitation procedure using weekly reports summarizing the number of steps per day, average number of steps per week, minutes spent performing low-, medium-, and high-intensity activity per day, and other auxiliary data. Numbers of steps per minute were used to estimate low-intensity activity (< 80 steps), medium-intensity activity (80-110 steps), and high-intensity activity (>110 steps). In addition to this weekly report, the system generated general time period reports as well as a complete report of data divided into 1-min intervals for further medical analyses.

**Fig. 2.**
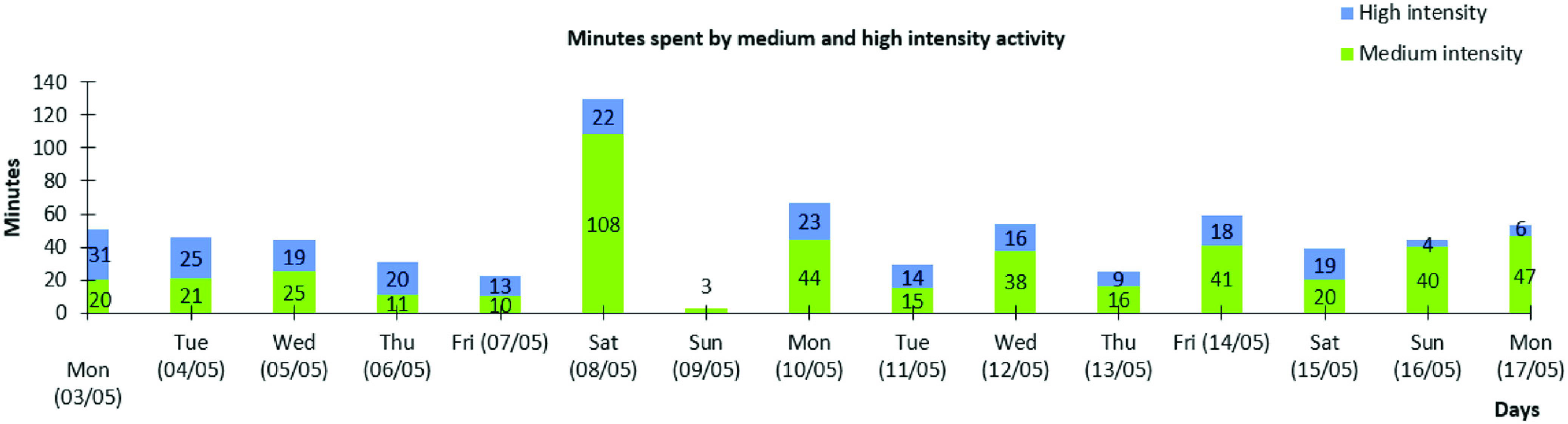
Example graph from a patient’s 14-day report showing minutes spent in medium- and high-intensity physical activity.

All reports were generated from the back-end system administrative Web app and downloaded in the MS Excel (.xlsx) format, including graphs visualizing number of steps per day and number of minutes spent in medium- and high-intensity physical activity per day. An example of a graph from a patient’s 14-day report is presented in Fig. 2.

### C. Connector Mobile Application

The Gadgetbridge app (see Fig. 1) is a publicly available freeware application and is documented on its project pages. Hence, this section describes the Connector app developed by the project team. The Connector app was installed on the patient’s mobile phone together with the Gadgetbridge app and allowed collected data to be transmitted directly to the server’s back-end application.

**Fig. 3.**
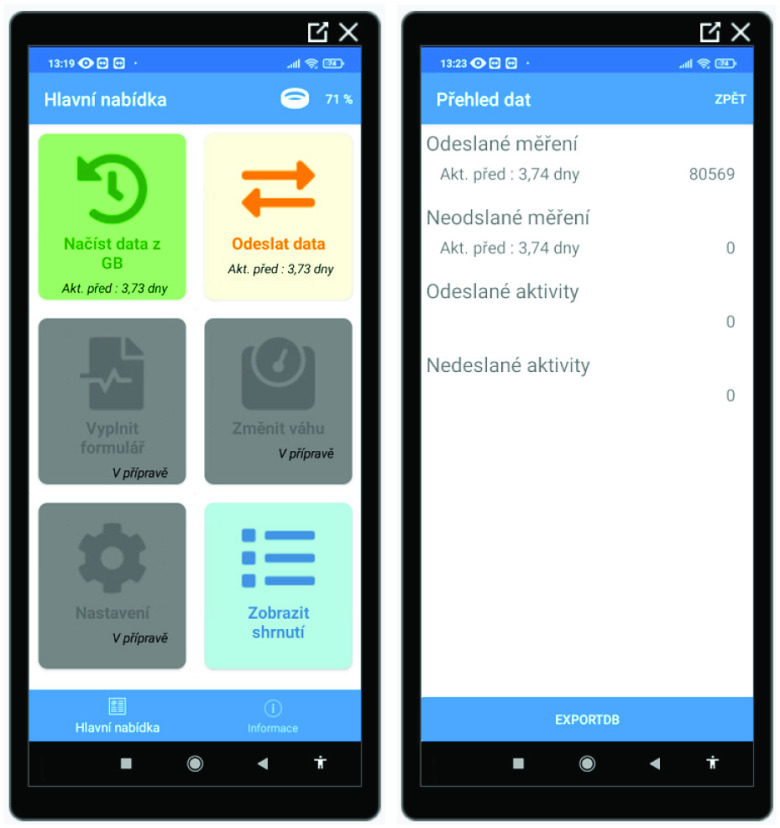
Example of the user interface in the patients’ Connector app (in Czech).

Fig. 3 presents two examples of the Connector app user interface. The left-side image depicts the main user menu, whereas the right side shows an overview of data synchronization status with the server’s back-end application.

The Connector app automatically synchronized the data from the Gadgetbridge Transfer Database with the back-end application each hour. In the case of synchronization failure or if data needed to be synced within a shorter period of time, syncing could be done manually via two buttons in the main Connector app menu: to load data from the Gadgetbridge app and send them to the back-end app.

### D. Data Transfer

As patient data collection and storage are key to telerehabilitation and telecoaching, we provide additional details regarding the data transfer procedure in this section.

Data from patient Wristbands were transmitted via Bluetooth to the Gadgetbridge app in the Xiaomi proprietary format. The transmission between the Gadgetbridge and Connector apps used the SQLite database file (DB3 format) created by the Gadgetbridge app for local file storage on the device.

The Connector app then sent the data to the back-end app using the standard Internet HTTPS protocol. The transfer was performed via a REST application programming interface (API) provided by the back-end app. Documentation of this API is available as online supplementary material.^2^ The API immediately saved the received data to the back-end database, and the process was accompanied by a logging mechanism, allowing for fast solutions to possible issues.^2^https://still.felk.cvut.cz/teresa/Teresa_BE_API_documentation.pdf, also https://doi.org/10.21227/z36r-0m98

For a more detailed evaluation, we provide anonymous details of the Wristband protocols as an online supplementary material.^3^ A description of the protocol is part of the supplementary material. In the protocol details, we have removed the device’s MAC address. Only, anonymous participant ID is used to group the data.^3^https://still.felk.cvut.cz/teresa/Teresa_wristband_protocols.xlsx, also https://doi.org/10.21227/z36r-0m98

The Connector app bundled 50 data records per transmission to optimize network traffic and transactional data transfer. One data record corresponded to data from a 1-min period from one patient. The security aspects of this communication are discussed further in Section IV-F.

### E. Overcoming Device Compatibility Issues

The configuration used in the study led to a wide spectrum of device compatibility issues from the very start of system testing. These issues involved the following factors, individually or in combination.

1) Android operating system version.
2) Mobile phone manufacturer.
3) Individual security and privacy settings on patients’ mobile phones.

Regarding the first issue, for example, Samsung devices using Android version 9 had difficulties correctly rendering the Connector app user interface. In ASUS devices using Android version 7, communication between the Gadgetbridge and Connector apps was disrupted, and Samsung devices using Android version 11 had issues with the Wristband’s connection to the Gadgetbridge app in two separate instances.

The effort needed to overcome these issues created a significant burden for the project; hence, a pragmatic solution to issue the same mobile phone with Wristbands to patients was a more viable option economically. We used the Xiaomi RedMi 9A phone during the pilot phase of the project.

### F. Security and Privacy

When the Connector app was installed on the patient’s mobile phone, the MAC address of the Wristband was used to connect the app to Gadgetbridge. During this process, a unique, secure token for the Wristband was generated, which was later used by the server back-end app to authorize the Connector app.

To prevent forged data from being added to the server database, the following protocol was executed for each data transfer: 1) the secure token was analyzed and verified by the back-end app; 2) if the token was valid, the metadata of the device corresponding to the token were loaded; 3) if the device was known to the system (based on a comparison of the device’s actual MAC address and the MAC address from the device record) and the device was allowed by the back-end application, the data were saved to the database. At the network level, the HTTPS protocol was used for data transfer.

Patient data privacy was ensured in a straightforward manner. Only particular Wristbands were identified in the system, and no link between the patient and the corresponding Wristband was stored; these links were kept separately by the medical staff using the data.

## V. Pilot Study Design and Evaluation

The proposed system was verified in a pilot study with 14 patients conducted from April 2021 to June 2021 (including initial tests). In this study, patient data were collected consecutively for 35 days. This section presents the study’s design and results.

### A. Pilot Study Design

*Study Design:* This study was a single-center, parallel-group, randomized, controlled pilot study.

*Participants:* Fourteen patients with post-acute COVID-19 (seven weeks after COVID-19 onset; ten women and four men; mean age 48.7 ± 7.8 years; mean body mass index 31.2 ± 6.1 kg/m ^2^) without orthopedic, neurological, musculoskeletal, cognitive, or cardiovascular disorders volunteered to participate in this study. Post-acute COVID-19 means symptoms are present beyond three weeks but less than 12 weeks after disease onset [2], [12].

They were recruited from the Post-COVID Care Center at University Hospital Hradec Kralove. The inclusion criteria were mild to moderate dyspnea (dyspnea score of 1 or 2 on the Modified British Medical Research Council Scale [mMRC]), presence of fatigue during activities of daily living and/or during ordinary physical activities, and ability to walk independently without assistive devices. Patients did not participate in another outpatient rehabilitation program simultaneously. Patients with severe or very severe dyspnea (mMRC score 3-4) were not included in this study; an outpatient rehabilitation program was recommended. Patients were informed regarding the study protocol, and they signed an informed consent form approved by the institutional ethics committee (reference number 202104 P19). Procedures were in accordance with the ethical standards for human experimentation in compliance with the 1964 Helsinki Declaration and its later amendments. Patients were randomly divided into two groups—1) the telerehabilitation (experimental) group and 2) educational (control) group (seven patients in each group; average age in the telerehabilitation group was 46.4 ± 8.8 years and 51.0 ± 12.3 years in the educational group). After a baseline assessment, both groups underwent the same initial education with a pulmonologist and started one-week physical activity monitoring. After one week, both groups had an educational lesson and 60 min of exercise and breathing training with a physiotherapist (initial rehabilitation lesson) and received a brochure with exercises for home training. The educational group continued unsupervised home exercise training only. They were instructed to perform breathing exercises, endurance, and strength training every day according to the list of exercises in their brochure. The telerehabilitation group performed the same exercises as the educational group but had a telerehabilitation lesson with telecoaching with a physiotherapist once per week (as the experimental intervention). Both groups were instructed to contact the pulmonologist or the physiotherapist immediately if their condition worsened or new symptoms appeared.

*Structure of Telerehabilitation With Telecoaching:* Telerehabilitation lessons with a physiotherapist were conducted once per week. These lessons were supervised by the physiotherapist through video calls using the Zoom platform. After the baseline assessment, the physiotherapist determined the initial exercise types and intensity during the face-to-face rehabilitation lesson. Each telerehabilitation lesson lasted 40 min and consisted of education (5 min), breathing exercises, strength training and muscle stretching (20 min), relaxation (5 min), and telecoaching (5 min). At the end of each lesson, there was time for patient questions (5 min). The same physiotherapist led each lesson for all patients. The physiotherapist checked that the patient performed the exercises correctly and discussed how active the patient had been in the previous week and how to increase physical activity in the following week.

*Assessments:* Respiratory muscle strength, functional exercise capacity, dyspnea severity, depression, number of steps, and intensity of physical activity were measured at the baseline and after five weeks (post-treatment).

*The respiratory muscle strength test* was administered in accordance with guidelines from the American Thoracic Society and the European Respiratory Society [25]. Maximal inspiratory ( $PI_{\max }$) and expiratory ( $PE_{\max }$) mouth pressures were measured (Geratherm, Germany). The predicted values of both parameters were calculated according to the equations for men and women separately [26].

*Functional exercise capacity* was determined with the 6-min walk test (6MWT) administered in accordance with guidelines from the European Respiratory Society and the American Thoracic Society [27]. The 6-min walking distance (6MWD) was recorded in meters. The test was performed on a 30-m flat interior corridor. The predicted values were calculated according to the equations for men and women separately [28].

*Dyspnea* was assessed with the five-point mMRC [29]. Patients circled the grade of dyspnea (0-4) that most closely matched their breathlessness. Higher scores represented more pronounced breathlessness.

*Depression* was evaluated using the Zung self-rating depression scale (SDS). The self-administered survey contains 20 items. The raw score obtained from the survey is converted to a 100-point scale, the SDS index, which ranges from 25 to 100. A score between 25 and 49 represents a normal range, 50–59 is associated with mild depression, 60–69 with moderate depression, and 70 and above with severe depression [30].

*Physical activity* was measured daily using the Wristband’s triaxial accelerometer, which measured the number of daily steps and intensity of performed physical activity. The level of physical activity was measured daily for five weeks. The Wristband had to be worn on the left wrist for the entire day and night and could be removed only during a shower or bath.

*Statistical Analysis:* Statistical processing of measured values was performed using Statistica 13.4 (StatSoft, Inc., Tulsa, OK, USA). Basic descriptive statistics were then calculated, and the Wilcoxon Signed Rank Test and Mann-Whitney U test were used because of noncompliance with the condition of normality. Categorical parameters were described using an absolute (relative) frequency. The statistical significance level was set at $\alpha = 0.05$.

### B. Results

**Fig. 4.**
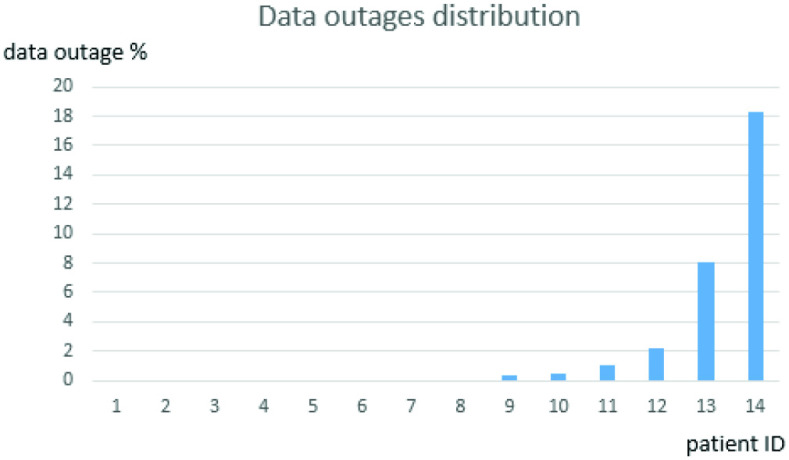
Independent-samples median test for difference in steps.

**Fig. 5.**
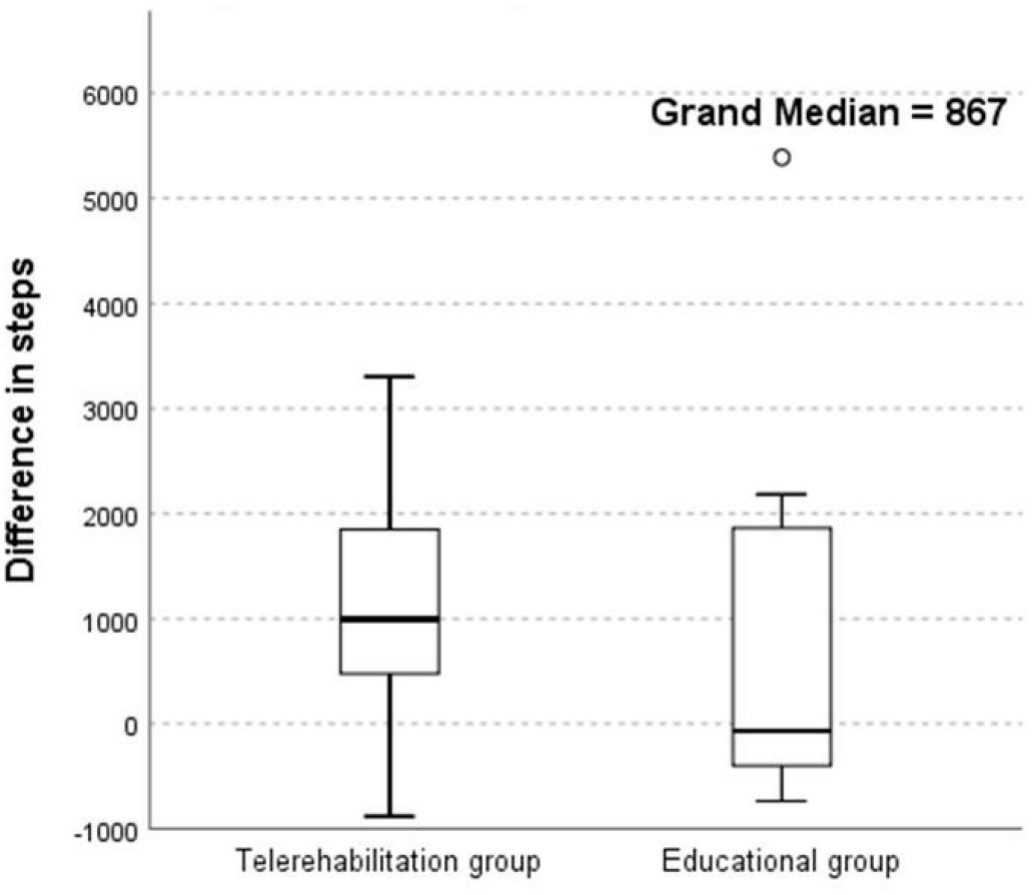
Independent-samples median test for difference in the 6-min walk test.

In this pilot study, we found that weekly telerehabilitation with telecoaching supervised by a physiotherapist led to an increase in physical activity (number of steps per day) in 85.7% of patients, while in the educational group, there was an increase in physical activity in 41.9% of patients (Table I and Fig. 4). Most physical activity was performed at low intensity in both groups, and physical activity intensity was not influenced by the chosen intervention in either group. Exercise capacity improved in 71.4% of patients in the telerehabilitation group and in 42.8% of patients in the educational group (Fig. 5). A significant increase in inspiratory muscle strength was observed in both groups. Only one patient in the telerehabilitation group and one in the educational group reached more than 100% of the predicted normal value at the baseline. On the contrary, 42.9% of patients in the telerehabilitation group had their values of inspiratory muscle strength lower than 70% of the predicted normal value and 71.4% of patients in the educational group at baseline. Post-treatment, three patients in the telerehabilitation group and two patients in the educational group had values higher than 100% of the predicted normal value. Decreased inspiratory muscle strength (lower than 70%) remained in 14.3% of patients in the telerehabilitation group and in none of the patients in the educational group after the treatment. Expiratory muscle strength increased in 71.4% of patients in both groups. The severity of dyspnea decreased in 57.2% of patients in the telerehabilitation group ( $p = 0.069$) and in 42.8% of patients in the educational group ( $p = 0.109$). At the baseline, four patients in the telerehabilitation group and two patients in the educational group were mildly depressed. The SDS index significantly decreased in both groups after treatment, but two patients in the telerehabilitation group and one patient in the educational group remained mildly depressed. The detailed results are listed in Table I.

**TABLE I:** Outcome Changes for Patients Receiving Education With the Home Exercise Program Versus Those Receiving Education With Telerehabilitation Including Telecoaching

Variable	Baseline	Follow-up	Within-group Median (IQR)	p value^1^	Between-group diff. Median (IQR)	p value^2^
Steps per day						
EG	5870	7101	994 (475.0 ÷ 1848.0)	.063	867 (−180.1 ÷ 1855.5)	1
CG	7181	7125	−70.3 (−400.6 - 1861.6)	.499		
PA_LA						
EG	1420	1400	−3 (−20.3 ÷ −1.4)	.176	−9.7 (−34.2 ÷ −1.9)	.286
CG	1415	1408	−13,7 (−33,7 ÷ −9,7)	.028		
PA_MA						
EG	19.1	19.0	3.2 (0.8 ÷ 3.8)	.237	2.6 (−0.03 ÷ 3.8)	1
CG	20.4	19.6	2 (−2.9 ÷ 3.6)	.612		
PA_HA						
EG	1.7	2.9	−0.1 (−0.2 ÷ 2.4)	.612	−0.05 (−0.2 ÷ 2.0)	1
CG	3.1	4.4	0 (−1.8 ÷ 1.7)	.917		
6MWD (% of predicted)						
EG	84.9	90.4	11.5 (1.4 ÷ 14.1)	.075	1.4 (0 ÷ 12.0)	.286
CG	87.2	87.2	0 (0 ÷ 4.8)	.180		
PI_max_ (% of predicted)						
EG	87.6	97.9	16.5 (13.7 ÷ 24.8)	.018	19.4 (12.3 ÷ 26.2)	1
CG	66.3	83.3	22.3 (10.6 ÷ 25.7)	.028		
PE_max_ (% of predicted)						
EG	84.0	81.7	8.8 (−1.5 ÷ 29.3)	.236	16.3 (1.9 ÷ 32.8)	1
CG	59.2	76.2	23.1 (4.8 ÷ 33.9)	.075		
SDS index						
EG	55	42	−5.0 (−9.0 ÷ −3.5)	.018	−6.5 (−11.0 ÷ −5.0)	.286
CG	43	27	−8.0 (−10.0 ÷ −6.5)	.018		

Notes: EG–experimental (telerehabilitation) group, CG–control (educational) group; PA_LA: low intensity of physical activity (minutes per day); PA_MA: medium intensity of physical activity (minutes per day); PA_HA: high intensity of physical activity (minutes per day); 6MWD: 6-min walking distance; PI_max_: maximal inspiratory mouth pressure; PE_max_: maximal expiratory mouth pressure; SDS index: Zung Self-Rating Depression Scale index;

^1^ Related-Samples Wilcoxon Signed Rank Test,

^2^ Mann-Whitney U test

### C. Data Collection Reliability

Correct transmission of data was vital in the system used in this study; hence, we outline our testing strategy that can be applied to create a similar type of system. During data transfer, data unreliability might be generated in two main ways.

1) Connectivity or data transfer outages in the pipeline from the patient’s Wristband to the back-end server.
2) Inaccuracy of the data as measured and transmitted from the patient’s Wristband, which typically depends on the accuracy of the sensors or computational and data transfer algorithms within the personal device.

The first issue was addressed by system testing with the project team and the pilot run with the initial group of patients. We conducted the first round of reliability tests with 11 participants from the project team one month before the start of the pilot study. Additionally, we conducted other tests to verify data was being transferred correctly with the first six participants from the pilot study group. We applied three main tests and subsequent user-based beta testing in this process.

First, we thoroughly tested all data transfer links between individual components of the system (see Fig. 1) using unit tests to verify the correct transfer of the data.

Second, in several end-to-end (E2E) test rounds with different system configurations, based on workflow testing techniques [31], [32], we evaluated all data records present on the back-end server. In this analysis, we distinguished situations when the data record was completely missing and was present, but the data were not matching tested situation.

Third, we examined aggregate data stored in the server’s back-end database (e.g., total number of steps per day) and compared them to the data displayed on the personal device.

Fourth, individual data transfer issues were tracked and investigated during the pilot run with the initial group of patients. Of all the issues reported, all were caused by connectivity problems rather than leftover software defects in the system.

The second issue was addressed by a long-term evaluation of data collection reliability. The 14 patients in the pilot study were monitored for 35 days, of which seven were an initial monitoring period; thus, the data collected during the subsequent 28 days were used by the medical and physiotherapy staff.

Individuals’ data records were broken down into one-min intervals, and a total of 690 359 1-min records were collected during the 35-day period. The expected data volume for one patient was 50 400 1-min records per monitored period, which was reached for eight patients. For the remaining six patients, the collection process was affected by various data collection outages, described as follows.

**Fig. 6.**
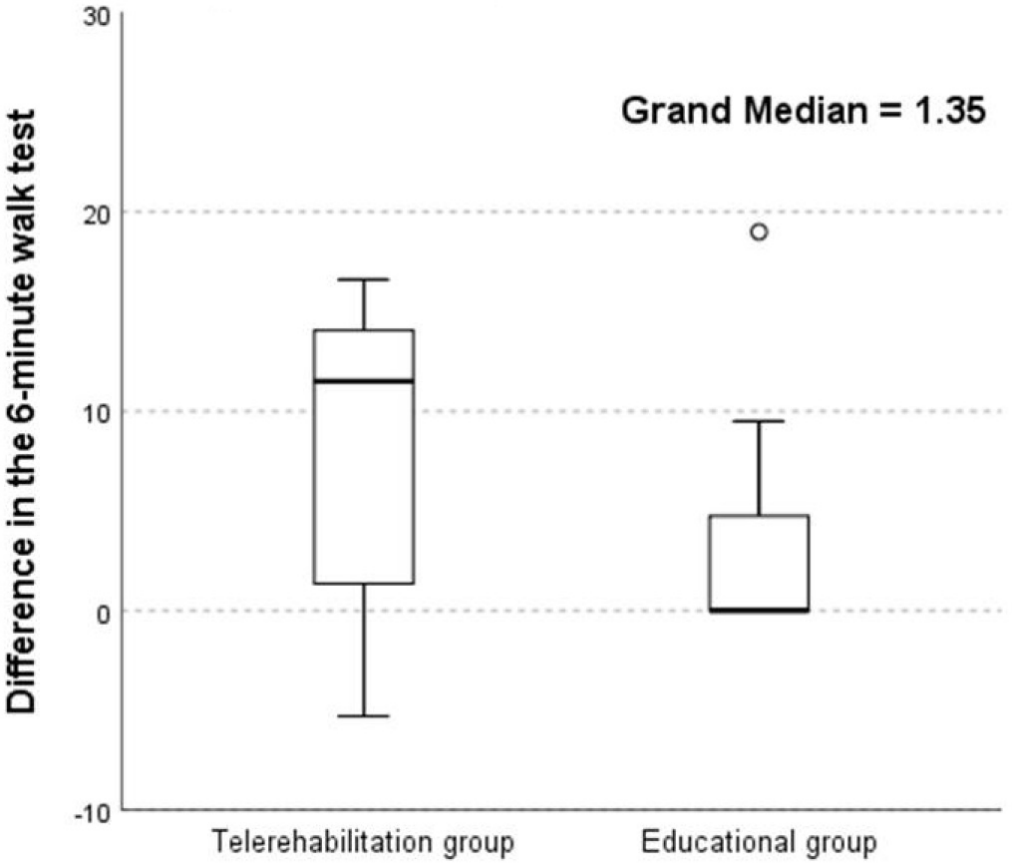
Distribution of data outages among individual patients.

The overall data volume collected for one patient in the 35-day period was 49 311 1-min records. The overall outage for the entire group was 2.2% of data records. For the six patients with missing data, the smallest outage was 0.3% and the largest 18.3%. The distribution of outages is shown in Fig. 6.

The majority of analyzed outages corresponded to two patients: IDs 13 and 14 in Fig. 6. We analyzed all outage cases and were in contact with the patients to restore data collection. Almost all outages were due to the patient not wearing the Wristband for some period or Bluetooth disconnection between the patient’s Wristband and the mobile phone. We identified no outage caused by a system software defect or outage in the GSM connection between the patient’s mobile phone and the back-end server.

As similar situations could occur in future projects, we further analyzed the reasons for data loss. In the case of patient ID 13, Bluetooth connectivity between the Wristband and the mobile phone was periodically disrupted by other Bluetooth and Wi-Fi-transmitting devices in her living room (smart TV and home Wi-Fi network). This problem was fixed when the Wristband and mobile phone were swapped for different hardware, and the patient kept her mobile phone in other parts of the room. Because the patient was located far from technical support, these outages affected 6.5 days of data collection.

Patient ID 14 reported she lost her Wristband when gardening and was unable to find it before night fell. The Wristband was replaced, and the connection was restored after two days and 7 h. The rest of the patients who experienced some data outage (IDs 9–12) had removed the Wristband for a short period (mostly to sleep or bathe) and forgot to wear it again.

**Fig. 7.**
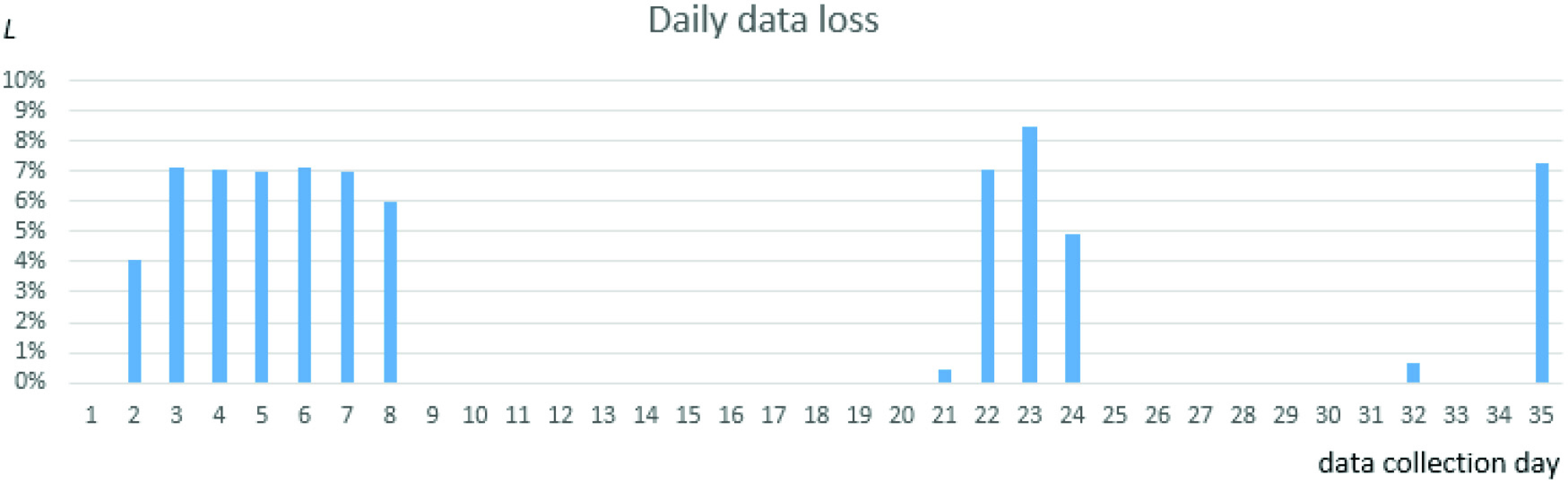
Distribution of data outages over the pilot study period.

Regarding the distribution of data outages over time, out of the 35 days, data collection outages (i.e., some missing data in the period) were recorded for 13 days. In one day, 20 160 1-min data records were expected for all 14 patients, denoted as $E$. Fig. 7 presents the percentage of data loss $L = (1 - {N}/{E}) \cdot 100\%$, where $N$ denotes the number of 1-min data records actually collected on the given day.

However, the fact that a 1-min data record was successfully saved in the system’s back-end database did not mean that the collected data regarding a patient’s physical activity were reliable. We further analyzed physical activity-related information using data collected concerning the patient’s heart rate.

**Fig. 8.**
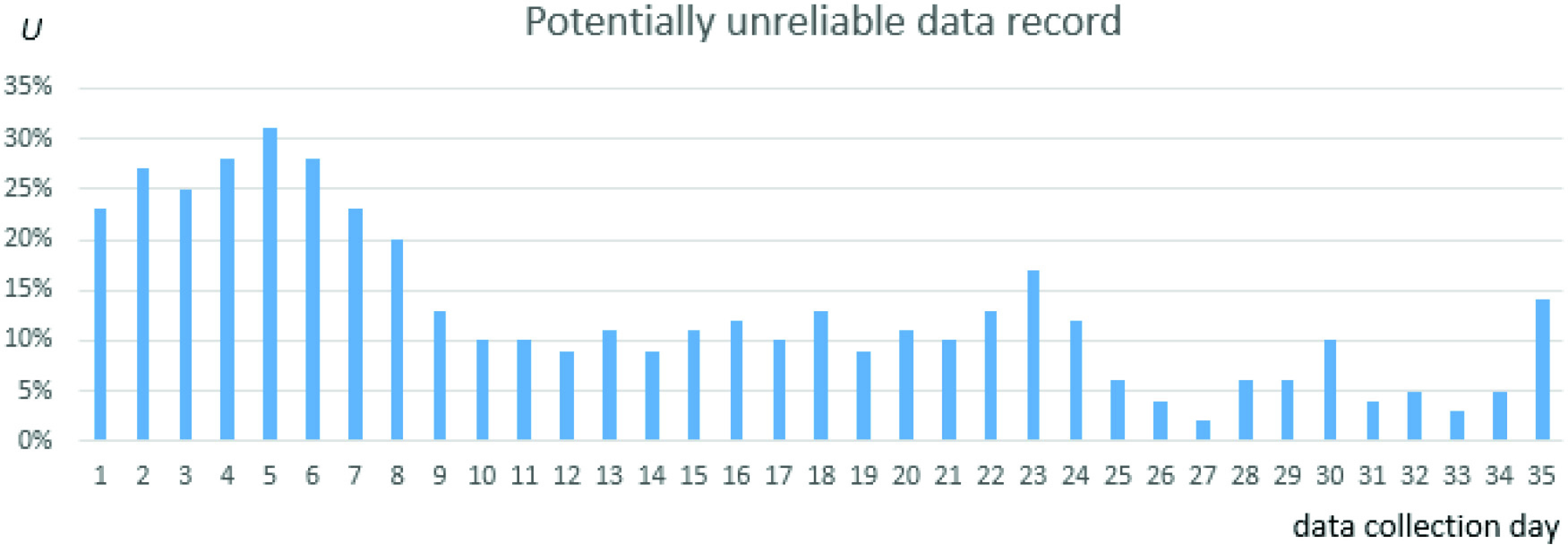
Distribution of potentially unreliable data records over the pilot study period.

In some cases, especially at the beginning of the study (the seven days of the initial monitoring period), patients needed time to become familiar with using the Wristband; hence, the heart rate sensor was not always in proper contact with the patient’s skin. The extent of this effect is shown in Fig. 8 and is expressed as a percentage of the collected data records for which the heart rate sensor was not in direct contact with the patient’s skin, denoted as $U$.

This implies that the information regarding patient physical activity could be, to some extent, inaccurate. Two main reasons are possible: 1) the patient was wearing the Wristband, but skin contact was insufficient, so the collected physical activity data were not significantly affected or 2) the patient was not wearing the Wristband at the moment, but the Wristband itself was moving. For example, during bathing, the patient put the Wristband somewhere in the bathroom, or the Wristband was in a bag being carried by the patient. The second case could have led to some bias in the collected data regarding the patient’s physical activity, but it is impossible to distinguish the first from the second situation.

The average $U$ for the entire monitoring period was 12.9%, dropping from 26.4% on average for the first seven days to 6.7% on average for the last seven days. Fig. 8 shows how patients became more familiar with the Wristbands as time passed and started wearing them correctly.

During the study, one patient frequently refused to wear the Wristband tightly, as instructed by the manufacturer, to allow for correct heart rate data collection; the Wristband caused a skin reaction and considerable discomfort. Considering the data, more patients in the first three weeks of the data collection period were not wearing the wristband properly.

No significant difference in data collection reliability was observed between the telerehabilitation and educational groups. In particular, the difference in the total collected 1-min records in both groups was 4.8%, which was statistically insignificant.

### D. Accuracy of Data Produced by Used Wristbands

Apart from data collection reliability, another aspect to consider is the accuracy of Wristband data. To test accuracy, we compared physical activity intensity outputs from the Wristband and the research-grade ActiGraph wGT3X-BT (ActiGraph LLC, Pensacola, FL, USA), hereafter referred to as “ActiGraph.” The ActiGraph accelerometer is well validated [33] and has been extensively used for evaluating physical activity [34], [35]. Under laboratory conditions, two participants wore both devices on their nondominant wrist and performed a walking/running protocol for a total duration of 57 min, which consisted of the following sequence.

1) Walk 1 km/h, 7 min.
2) Sedentary activity, 5 min.
3) Walk 2 km/h, 5 min.
4) Sedentary activity, 5 min.
5) Walk 4 km/h, 6 min.
6) Sedentary activity, 6 min.
7) Walk 6 km/h, 6 min.
8) Sedentary activity, 5 min.
9) Run 8 km/h, 6 min.
10) Sedentary activity, 6 min.

A treadmill with a set speed was used to maintain the walking and running pace. Regarding physical activity intensity output, both devices differed in scale. The Wristband produced a value ranging from 0 to 255 (no unit), whereas the ActiGraph measured intensity in milligravitational units ( $\text{m}{g}$), ranging from 0 to 8900 mg. Given that the ActiGraph was initialized to collect data at 100 Hz, the output data were subsequently reintegrated into a 1-min epoch to be comparable with the 1-min output from the Wristband. We standardized the outputs from both devices using $z$-scores.

As the data did not meet the condition of normality, we used the Mann-Whitney U test for comparison. With the significance level set at p = 0.05, the difference of intensity data collected by the Wristband and ActiGraph was not statistically significant for either participant (s1 = 0.41 and s2 = 0.91).

**Fig. 9.**
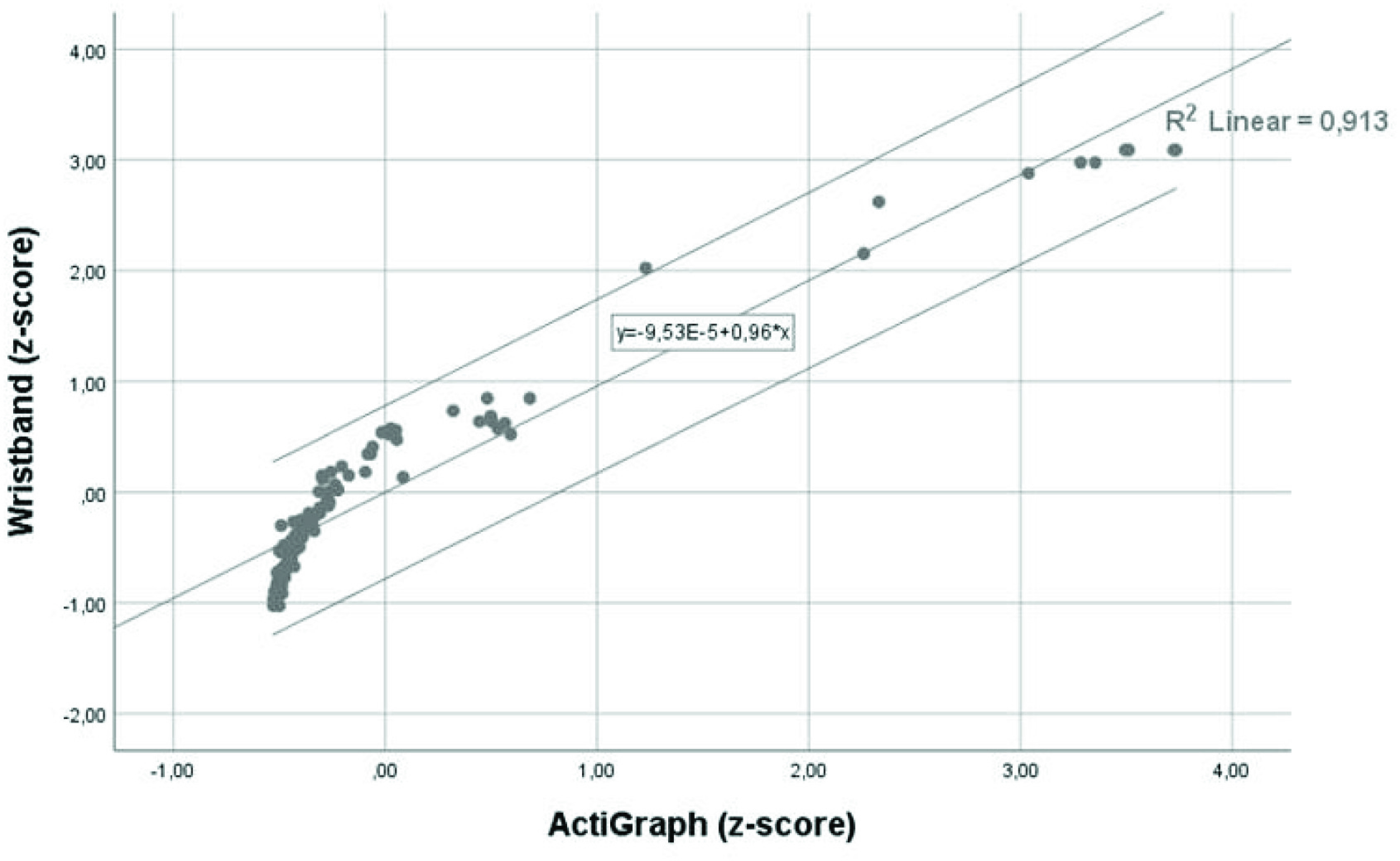
Correlation analysis to verify the nonstatistically significant difference between the results of Wristband and ActiGraph.

We have made a correlation analysis to verify the nonstatistically significant difference between the results of Wristband and ActiGraph. The value of the correlation coefficient was $R=0,955$ ( $R^{2}=0,913$) and is significant at 0.01 level. Details from this analysis are presented in Table II and Fig. 9.

**TABLE II:** Correlations Between Data From Wristband and ActiGraph

		Wristband (z-score)	ActiGraph (z-score)
Wristband (z-score)	Pearson Correlation	1	.955**
	Sig. (2-tailed)		.000
	N	114	114
ActiGraph (z-score)	Pearson Correlation	.955**	1
	Sig. (2-tailed)	.000	
	N	114	114

^**^ Correlation is significant at the 0.01 level (2-tailed).

**Fig. 10.**
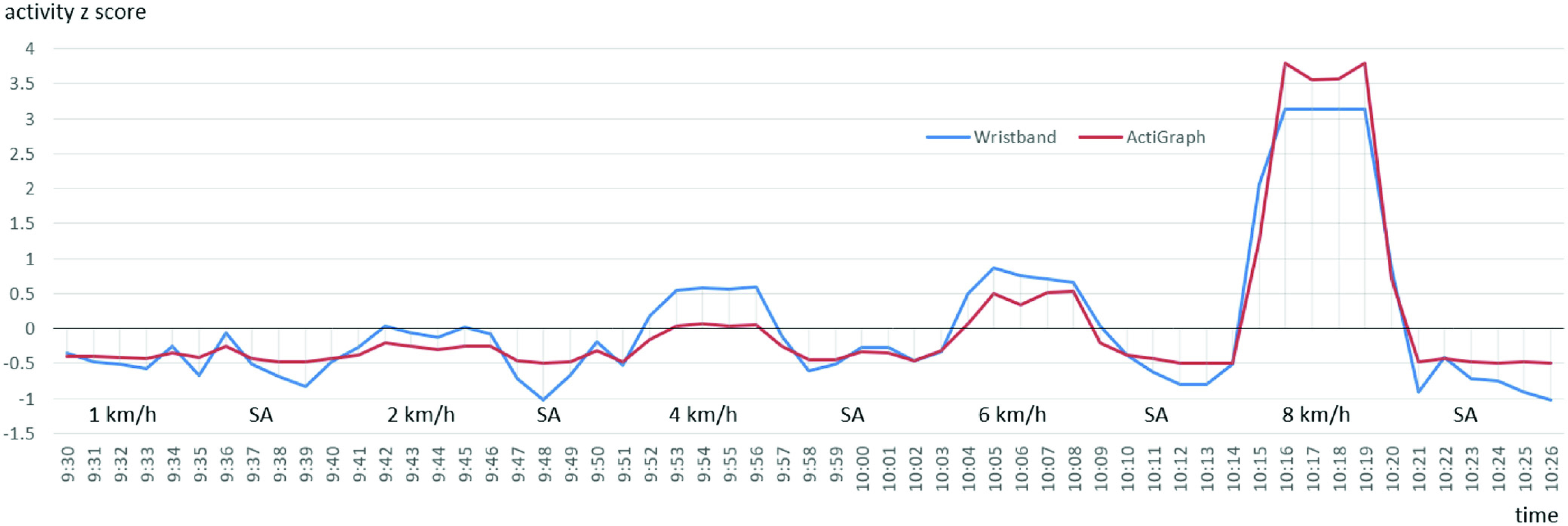
Activity $z$-score for the first participant who used the Wristband and ActiGraph simultaneously during the experiment.

**Fig. 11.**
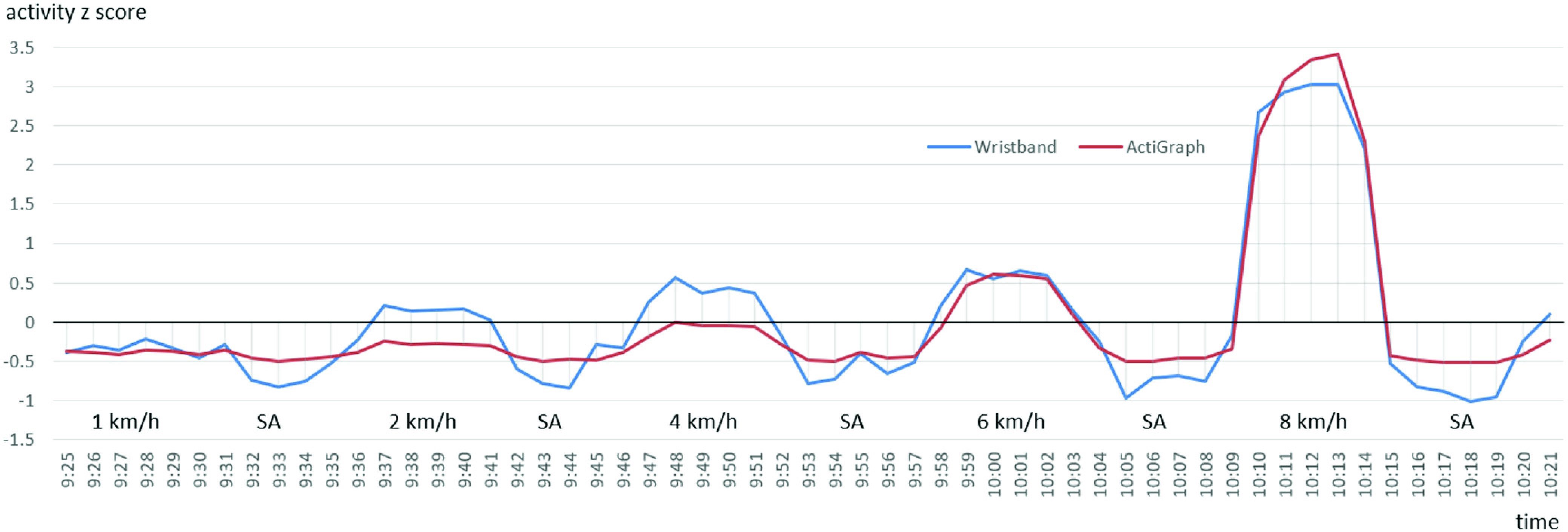
Activity $z$-score for the second participant who used the Wristband and ActiGraph simultaneously during the experiment.

Figs. 10 and 11 present the $z$-scores of the measured activity during the experiment period for both participants who used the Wristband and ActiGraph simultaneously. SA stands for sedentary activity. No clear trend can be observed for $z$-score peaks when comparing Wristband and ActiGraph data.

Hence, the Wristband can be considered sufficiently accurate for the telerehabilitation program examined in this study. However, this conclusion only applies to telerehabilitation as described in this study; this conclusion cannot be extended for other cases or medical use without a proper accuracy test.

## VI. Lessons Learned

Several unexpected issues occurred during this project’s rapid preparation and implementation, which led to extra work for the project team. As such obstacles might necessitate additional resources and even delay similar projects, we discuss them in the following section.

### A. Technical Lessons Learned

Technical takeaways from the project can be divided into three categories: 1) data privacy and access; 2) compatibility issues; and 3) technical limits of devices.

For *data privacy* reasons, we strongly preferred to not store patients’ data in a third-party cloud database, which in fact became the project’s greatest technical challenge. Commercial wearables are rarely designed to transmit data directly to a defined user interface; instead, data are commonly stored by the manufacturer and subsequently accessed through an API. In our project, we used the Gadgetbridge app, which solved this problem and allowed a direct connection between the device and the medical staff, even if several data transfer steps were necessary (see Fig. 1). This limitation should be considered when assessing the applicability of commercial wearables for similar projects.

Furthermore, we strongly recommend verifying compliance of the data privacy procedures with the actual EU or WHO legislation and recommendations.^4^, ^5^, ^6^, ^7^, ^8^^4^Directive 2002/58/EC concerning the processing of personal data and the protection of privacy in the electronic communications sector.^5^Regulation (EU) 2016/679 of the European Parliament and of the Council of 27 April 2016 on the protection of natural persons with regard to the processing of personal data and on the free movement of such data, and repealing Directive 95/46/EC (General Data Protection Regulation.)^6^Directive 2011/24/EU of the European Parliament and of the Council of 9 March 2011 on the application of patients’ rights in cross-border healthcare. Official Journal. 2011;L 88:45-65.^7^Decision 2019/1765 of 22 October 2019 Providing the rules for the establishment, the management and the functioning of the network of national authorities responsible for eHealth, and repealing Implementing Decision 2011/890/EU.^8^WHO/EURO:2021-1994-41749-57154 The protection of personal data in health information systems-principles and processes for public Health.

Regarding *access to collected data*, even when a standard model is employed and data are accessed from the device manufacturer’s storage location, we strongly recommend performing a proof of concept regarding how access rights to the data are granted, if any restrictions exist, and how the manufacturer’s technical support responds to access requests. Unfortunately, there is no general guarantee that the manufacturer will grant the API access to the cumulative data.

Several *device compatibility issues* were already discussed in Section IV-E. The major issue is the variety of mobile phones and their operating systems among patients. Considering a larger group of patients of various ages and income levels, even relatively obsolete mobile phones may still be found in the group. To mitigate this issue, thorough compatibility testing must be performed before the system is used with patients.

During the pilot project, we also experienced Bluetooth *connectivity issues* between patients’ Wristbands and mobile phones. Even if the Wristband was properly paired with the mobile phone, in one case, home Wi-Fi and a Bluetooth signal from a smart TV interfered with the Bluetooth signal between the Wristband and the phone, leading to connectivity outages. This situation might potentially occur more frequently when the system is used for a larger group of patients, some of whom may have various types of smart home networks in their homes. Technical support is needed for such cases, which we discuss in Section VI-B.

Another issue is the *durability* of the Wristband and *user comfort*. When wearables do not belong to particular patients, the hardware might be treated with less care, especially charging connectors. A magnetic connector is preferred over the standard USB connector. In addition, low user comfort might lead to lower acceptance of the wearable by patients and ultimately low willingness to use the wearable consistently, which would impact data collection reliability. Furthermore, skin reactions caused by the Wristband material were experienced in two cases; however, to collect accurate data, the Wristband sensors must maintain contact with the patient’s skin for an extensive period. We recommend testing such user aspects during a simple proof of concept before the actual distribution of wearables to patients.

### B. Organizational Lessons Learned

We also determined some guidelines related to organizational issues, which can be grouped into two major categories: 1) technical support for patients and 2) issues arising from the distribution of extra hardware to patients.

A detailed *user manual* that includes technical support information is essential for patients. It should consider their diverse backgrounds and ages, as well as their physical state after the disease. However, even a very detailed manual was considered insufficient in our project, as more intense support was necessary.

During pilot study enrollment, the Wristband handover and initial *user training* were part of the entrance medical examination of the patients included in the study. Patients were instructed on how to use the Wristband and apps, mainly how to keep the Wristband connected to the issued mobile phone and how to sync the collected data. To save time during the exam, it was essential to have *installed* Wristbands and mobile phones. This preparation included the preinstallation of the Gadgetbridge and Connector apps, connection of all components, registration of the Wristband on the server’s back-end app, and proper data transmission testing.

In addition to the initial user training, a dedicated person for *user support* was required to solve various connectivity issues during the pilot project. To communicate with patients, their personal mobile phone numbers were given to the person in charge of technical support. If the data were not synchronized to the server for a period longer than one day, technical support contacted the patient to determine the possible cause and fix the synchronization issue. To minimize the need to meet in person with the patients, a *remote control* application (Team Viewer) was installed on the mobile phones provided to the patients. Only in cases where it was not feasible to solve the issue via phone call and the remote control program was an in-person meeting between the patient and tech support arranged.

Some further issues, which may be obvious to a typical user of information technologies, occurred in our project. Regular *charging* of the Wristband and issued mobile phone was necessary, and the patients were instructed to do so periodically. In addition, a plan in case a patient lost the Wristband was needed, which happened several times during the pilot study. Contingency hardware should be available for such cases, and data collection continuity must be maintained when hardware is replaced during the data collection period.

Another issue that we did not foresee was the necessity of wearing the Wristband so its sensor made *proper contact with the skin*. As discussed previously (see Fig. 8), this was not the case for all patients at the beginning of the study, and despite becoming less of a problem during the study, it was not entirely minimized.

The last issue involved patients not always taking the issued mobile phone with them when leaving their houses. To manage this, *delayed data synchronization* was configured, and when the data were not synchronized for a longer period (typically two days), user support called the patients to remind them to synchronize the data.

As this last point is crucial for a successful telerehabilitation program, we discuss it further. The physiotherapist consistently reviewed the recorded data before the telerehabilitation lesson so she could evaluate the physical activity of the patients in advance and determine whether they had performed sufficient physical activity. She evaluated the data individually for each patient and, based on the results, determined the target number of steps for the following week. However, as mentioned, not all patients uploaded the data in time, so the physiotherapist needed to communicate with technical support to determine why the patient’s data were missing. Without the missing data, it was impossible to accurately analyze and determine the level of physical activity for the following week. However, this occurred mainly at the beginning of the pilot study. Moreover, patients in the telerehabilitation group were reminded during each telerehabilitation lesson of the importance of recording data daily so an accurate analysis of their physical activity level could be made before the next meeting. The data obtained before the telerehabilitation lesson were beneficial for the therapist because she could evaluate them in detail and prepare for the online lesson with patients. Moreover, the therapist could discuss why the patient failed to perform sufficient physical activity on a particular day; the most common reasons were health problems, caring for a sick child, and a long trip by car.

## VII. Discussion and Threats to Validity

Based on the results of the pilot study, the proposed project is viable and will be further verified in another study with a larger group of patients: the TERESA 2 project (202111 P05) starting in February 2022. The telerehabilitation and telecoaching approach and created technical infrastructure are also applicable to patients with other respiratory diseases, such as COPD, bronchial asthma, interstitial lung disease, and other community-acquired pneumonia.

However, several issues may affect the validity of the results or influence the functionality of the proposed approach. This section discusses these issues along with mitigation actions and countermeasures to prevent them.

First, a more extensive controlled study should be conducted to provide more accurate data regarding the benefits of the telerehabilitation and telecoaching for patients with post-acute phase COVID-19. Our study was only focused on patients with mild to moderate dyspnea (dyspnea score of 1 or 2 on the mMRC); therefore, the results of this pilot study cannot be applied to patients with more severe dyspnea (mMRC score 3–4). Patients with more severe symptoms or patients with worse functional impairment after COVID-19 critical illness need an individualized and adapted inpatient or outpatient pulmonary rehabilitation program targeted to the needs of these patients [3]. Furthermore, systematic screening of patients at 6–8 weeks after COVID-19, as was also done in our study, is useful to identify patients with persistent symptoms [3]. It is necessary to prepare multidisciplinary care programs, including individually tailored pulmonary rehabilitation for these patients and especially for patients who were admitted to the intensive care unit because over a third of survivors who had critical COVID-19 can have a new disability in all areas of functioning in following six months [36]. Moreover, poor health can negatively influence their return to work [36].

However, the study presented in this article indicates the approach’s potential to improve patients’ physical activity, exercise tolerance (or capacity), and inspiratory muscle strength. Early disease-specific education on post-COVID conditions can positively influence patients’ functioning, and following telerehabilitation with telecoaching can improve the health status of patients with mild severity of their ongoing symptoms. As Thomas *et al.* [3] recommended, also patients in our study had been alerted for possible post-exertional symptom exacerbation and had been instructed on how to self-manage symptoms and when necessary to contact a pulmonologist or physiotherapist to minimize worsening of their health status during exercise training. Moreover, in future studies, it is necessary to evaluate the effect of telerehabilitation with telecoaching not only on the exercise tolerance, the daily level of physical activities, lung functions and respiratory muscle strength, symptom severity, and depression in patients with post-acute COVID but also to assess the effect of this treatment on patient’s health, functioning and his/her disabilities, quality of life or health-related quality of life, as is usually evaluated in other respiratory diseases. Standardized questionnaires or tools, which can be used for this evaluation, are, for example, the World Health Organization Disability Assessment Schedule 2.0 (WHODAS 2.0), the Euro Quality of life-five Dimensions-five Levels (EQ-5D-5L), or the St. George’s Respiratory Questionnaire (SGRQ) [36]–[38].

Persistent respiratory symptoms and/or long-lasting physical exercise intolerance can occur even after mild COVID-19 [39]. It is not entirely clear how patients’ rehabilitation activities post-COVID-19 should be timed. In theory, early rehabilitation care as soon as possible after acute respiratory problems have resolved would have a greater effect than a rehabilitation program begun later. All patients included in the TERESA study were in the post-acute phase of COVID-19, seven weeks after the confirmed diagnosis.

Regarding the accuracy of the data collected by the Wristbands, the comparison with the ActiGraph device (see Section V-D) showed that in the current study, the data accuracy from the commercial device was acceptable. However, this finding cannot be generalized to data different than those collected in this study or for other medical uses.

Data collection reliability is a major issue that could threaten the smooth operation of telecoaching and telerehabilitation. Longer data collection outages could make working with individual patients difficult for physiotherapists and potentially decrease telerehabilitation’s effectiveness. These outages must be prevented through a combination of data caching, as presented here, and the prevention of technical and organizational issues, as summarized in Section VI, including providing technical support ready to solve individual patient-side technical problems that might occur.

Possible disadvantages of Wristband use were outweighed by the favorable acquisition price of the devices and their good availability in the market. This is important, as it would facilitate the scaling-up of the project.

Regarding the great variety of commercial wristbands on the market, an effort to create a universal solution that would allow any device to connect to the system seems not worth the gained advantages. The acquisition price of the hardware was lower than the time the development team needed to create a completely interoperable and universal solution. However, we have currently added the Garmin and Fitbit wristbands to extend the compatibility of the system with market-available wristbands. We also plan to further increase compatibility for other brands with a significant market share.

Finally, many fitness wristbands are available that give feedback on physical activity level and tips for increasing it through a computer or smartphone program; however, these programs are designed for people who are already healthy. Thus, our proposed approach has the potential to expand physical activity support for people with health problems. In telecoaching applications in particular, therapists check and provide feedback on physical activity to patients, always taking into account their health condition so they can benefit as much as possible from the given telecoaching program. Extending and increasing the availability of existing telecoaching applications [5], [24] for routine daily clinical practice is therefore highly desirable.

Considering the fact that the size of the pilot study group is 14 patients, the goal of this article is to report on the technical feasibility of the concept and preliminary results that are encouraging for conducting a larger-scale pilot study which started in April 2022.

As this is the first controlled study of this kind, the results can be considered preliminary. We believe our results will be beneficial to those working to manage both the technical as well as medical aspects of post-acute COVID-19, as the situation requires rapid reaction, and knowledge-sharing in this sense is vital.

## VIII. Conclusion

The rapid pace of the project gave us a unique opportunity for on-the-ground learning, and we summarized all the gained technical and organizational knowledge in this article. We focused on lessons learned that might be useful for anyone in the technical and medical communities who are implementing a similar project or planning treatment for COVID-19 patients in the post-acute phase. As the medical evaluation of the pilot project shows, early disease-specific education on post-COVID conditions followed by telerehabilitation with telecoaching can improve patients’ physical condition in terms of physical activity, exercise tolerance, and inspiratory muscle strength.

Despite the availability of many mobile fitness applications and personal wearables, few exist with the capacity to directly support telerehabilitation and telecoaching programs, where medical staff involvement is needed. As such, low-cost commercial wristbands are essential to allow this concept to be scaled up for larger groups of patients. The smooth operation of the telerehabilitation program relies on patient data synchronization, and the importance of technical support cannot be underestimated.

This proposed approach can be applied to other pulmonary diseases, such as COPD, asthma bronchiale, interstitial lung disease, and other community-acquired pneumonia.
